# Amazon deforestation causes strong regional warming

**DOI:** 10.1073/pnas.2309123120

**Published:** 2023-10-30

**Authors:** Edward W. Butt, Jessica C. A. Baker, Francisco G. Silva Bezerra, Celso von Randow, Ana P. D. Aguiar, Dominick V. Spracklen

**Affiliations:** ^a^Institute for Climate and Atmospheric Science, School of Earth and Environment, University of Leeds, Leeds LS2 9JT, United Kingdom; ^b^INPE - Instituto Nacional de Pesquisas Espaciais, São José dos Campos 12227-010, Brazil; ^c^Stockholm Resilience Centre, Stockholm University, Stockholm 106 91, Sweden

**Keywords:** deforestation, temperature, climate

## Abstract

Tropical deforestation impacts the climate through complex land–atmosphere interactions causing local and regional warming. However, whilst the impacts of deforestation on local temperature are well understood, the regional (nonlocal) response is poorly quantified. Here, we used remote-sensed observations of forest loss and dry season land–surface temperature during the period 2001 to 2020 to demonstrate that deforestation of the Amazon caused strong warming at distances up to 100 km away from the forest loss. We apply a machine learning approach to show nonlocal warming due to forest loss at 2–100 km length scales increases the warming due to deforestation by more than a factor 4, from 0.16 K to 0.71 K for each 10-percentage points of forest loss. We estimate that rapid future deforestation under a strong inequality scenario could cause dry season warming of 0.96 K across Mato Grosso state in southern Brazil over the period 2020 to 2050. Reducing deforestation could reduce future warming caused by forest loss to 0.4 K. Our results demonstrate the contribution of tropical deforestation to regional climate warming and the potential for reduced deforestation to deliver regional climate adaptation and resilience with important implications for sustainable management of the Amazon.

## Significance

Tropical deforestation warms the climate with negative impacts on people living nearby. Most previous studies have focused on the local warming caused by deforestation and less is known about how deforestation impacts surrounding areas. Our study used satellite data to show that deforestation in the Amazon caused substantial warming up to 100 km away from the location of forest loss. We show that this nonlocal warming increased deforestation-induced warming by a factor of four. We estimate that reducing deforestation in the Brazilian Amazon could reduce future warming in the southern Amazon by 0.56 °C. These findings highlight the role of deforestation in regional climate change and emphasize the importance of reducing deforestation for climate adaptation and resilience in the Amazon.

Biophysical effects of forests can strongly impact properties of the land surface through changes in land–atmosphere fluxes of heat, moisture, and momentum (1–3). Forest landscapes typically exhibit higher leaf area index, deeper roots, lower albedo, greater evapotranspiration, and aerodynamic surface roughness compared to nonforest landscapes (1).

Changes to these biophysical effects as a result of deforestation cause changes in climate at the location of land cover change, known as local effects (4). Deforestation has contrasting local effects, with increased albedo acting to cool the surface whilst reduced surface roughness and evapotranspiration, acting to warm the surface (5). Numerous studies have shown that the combined impact of these contrasting effects is local warming with tropical deforestation increasing local daytime surface temperature by 1–2 °C or more (6–14). Deforested areas in the Amazon were found to have warmed by as much as 3 °C locally, with the largest warming during the dry season (15, 16). The local warming impacts of deforestation are well recognized by people living within tropical forest landscapes (17).

Deforestation can also impact the climate of nearby areas that are 10 s or 100 s of km from the location of land-use change (3). Much less is known about these nonlocal climate impacts of deforestation, which are more challenging to assess (18). Nonlocal impacts which occur via advection, circulation changes, and atmospheric feedbacks depend on the geographical distribution and spatial extent of surrounding deforestation, meaning that these effects are much more difficult to identify or interpret. Indeed, many observational assessments of the local impacts of land-use change use a “space-for-time” approach that compares the climate over forest and neighboring nonforest that excludes nonlocal effects or assumes that they are negligible (4, 19). In contrast, some observational studies suggest that nonlocal effects could be substantial. Large (>1,000 km^2^) deforested patches are known to warm more than smaller patches of deforestation (20). Cohn et al. (21) found significant and substantial nonlocal warming at undisturbed locations in Brazil’s Amazon and Cerrado biomes at distances up to 50 km away from forest loss. In maritime Southeast Asia, deforestation has been shown to result in warming up to 6 km away from the location of deforestation (22). Climate models have been used to separate the local and nonlocal effects of deforestation suggesting that the nonlocal effects of deforestation on surface temperature are similar in magnitude to the local effects (4). These studies have highlighted a potentially important, but poorly quantified, impact of deforestation on regional temperatures. A much better quantification and understanding of these regional responses is needed to inform sustainable management of tropical landscapes.

In this study, we examined both local and nonlocal effects due to forest loss across the Amazon biome where deforestation is dramatically changing the landscape (23). Large-scale deforestation started in the 1970 s, (24) and about 17% of the Amazon had been deforested by 2021, with rates of deforestation accelerating in the past few years (25, 26). A business-as-usual scenario of continued deforestation of the Amazon has estimated that up to 40% of the basin could be deforested by 2050 (27). Given the extent of land cover change across the Amazon, both local and nonlocal temperature responses to forest loss may represent an important mechanism for shaping local and regional climates.

We combined a number of remote-sensed datasets to quantify and predict changes in dry season surface temperature due to forest cover loss across the Amazon at varying length-scales. A previous study (21) assessed how warming in undisturbed areas of Amazon forest was impacted by the amount of nonlocal forest loss. Our study focuses on areas experiencing forest loss and aims to understand how temperature changes at the local scale depend on the extent of both local and nonlocal forest loss. We calculated the change in remotely sensed observations of land surface temperature at *n* = ~3.7 million locations (each 1 km^2^ in extent) across the Amazon between 2001 and 2020. Using remotely sensed observations of forest fraction over the same period, we explored how warming from forest loss depended on the extent of both local and nonlocal forest loss. We then used a machine learning approach to further isolate the local and nonlocal effects of forest loss. Finally, we use this model to make the first prediction of how the temperature response depends on local and nonlocal forest loss across the Amazon.

## Results and Discussion

### Forest Loss and Surface Temperature Change.

**Fig. 1.**
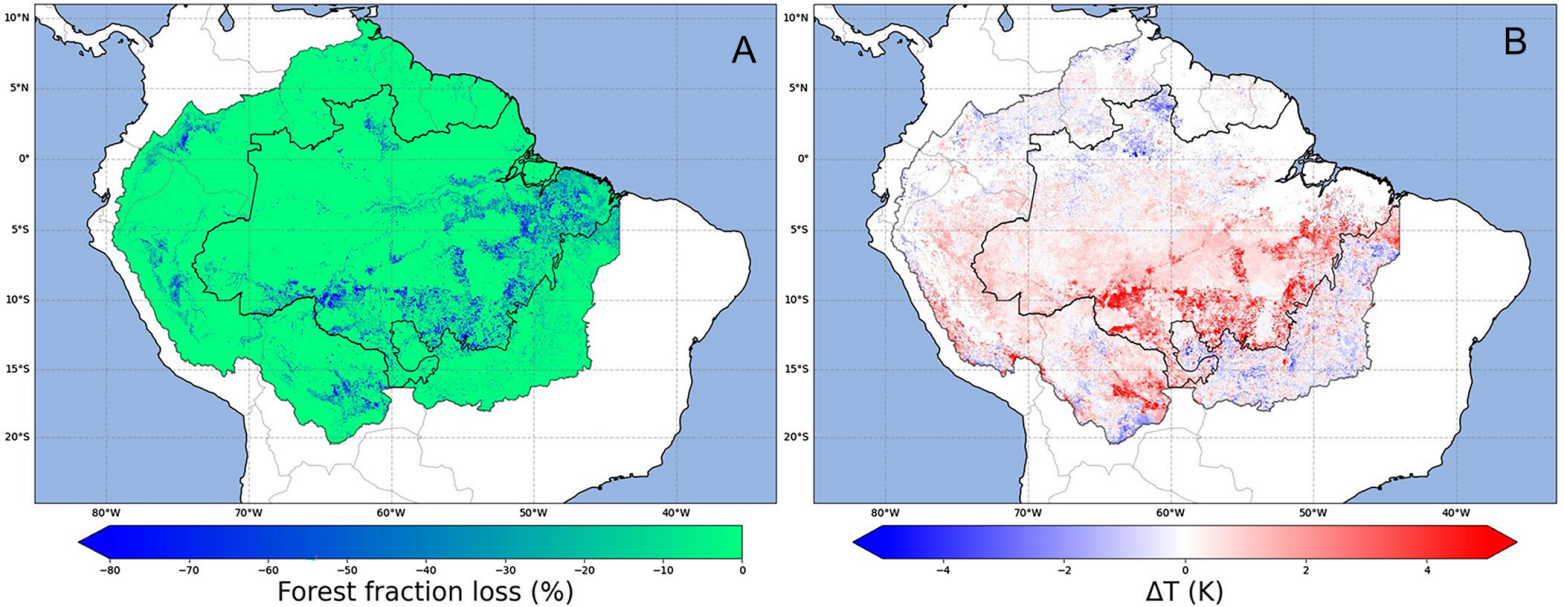
Forest loss and surface temperature change during 2001 to 2020. (*A*) Percentage point loss (%) in forest fraction. (*B*) Change in surface temperature (Δ*T*, Kelvin) of the driest month. Change in forest fraction and Δ*T* over 2001 to 2020 is calculated as the difference between the mean of the first 3-y and the last 3-y of the study period (i.e., 2001–2003 versus 2018–2020). Data are shown for the Amazon basin with the boundary of the Brazilian Amazon biome also shown.

Fig. 1 shows Amazon forest loss and corresponding dry season (defined as driest 3 mo per pixel) land surface temperature change (Δ*T*) over 2001 to 2020. Forest loss and Δ*T* show similar spatial patterns, with stronger warming over regions of forest loss particularly across the “arc of deforestation” along the southern Amazon. Warming exceeds 5 K over regions of extensive forest loss, with stronger warming over regions with more extensive forest loss (Fig. 1*B*). Across the whole Amazon basin (*n* = 3.7 million), median and mean Δ*T* were 0.48 and 0.6 K, respectively (Fig. 1*B*). Across the Brazilian Amazon biome, observed median and mean Δ*T* were greater at 0.58 and 0.78 K, respectively (Fig. 1*B*).

### Observed Δ*T* as a Function of Forest Loss at Local and Regional Scales.

**Fig. 2.**
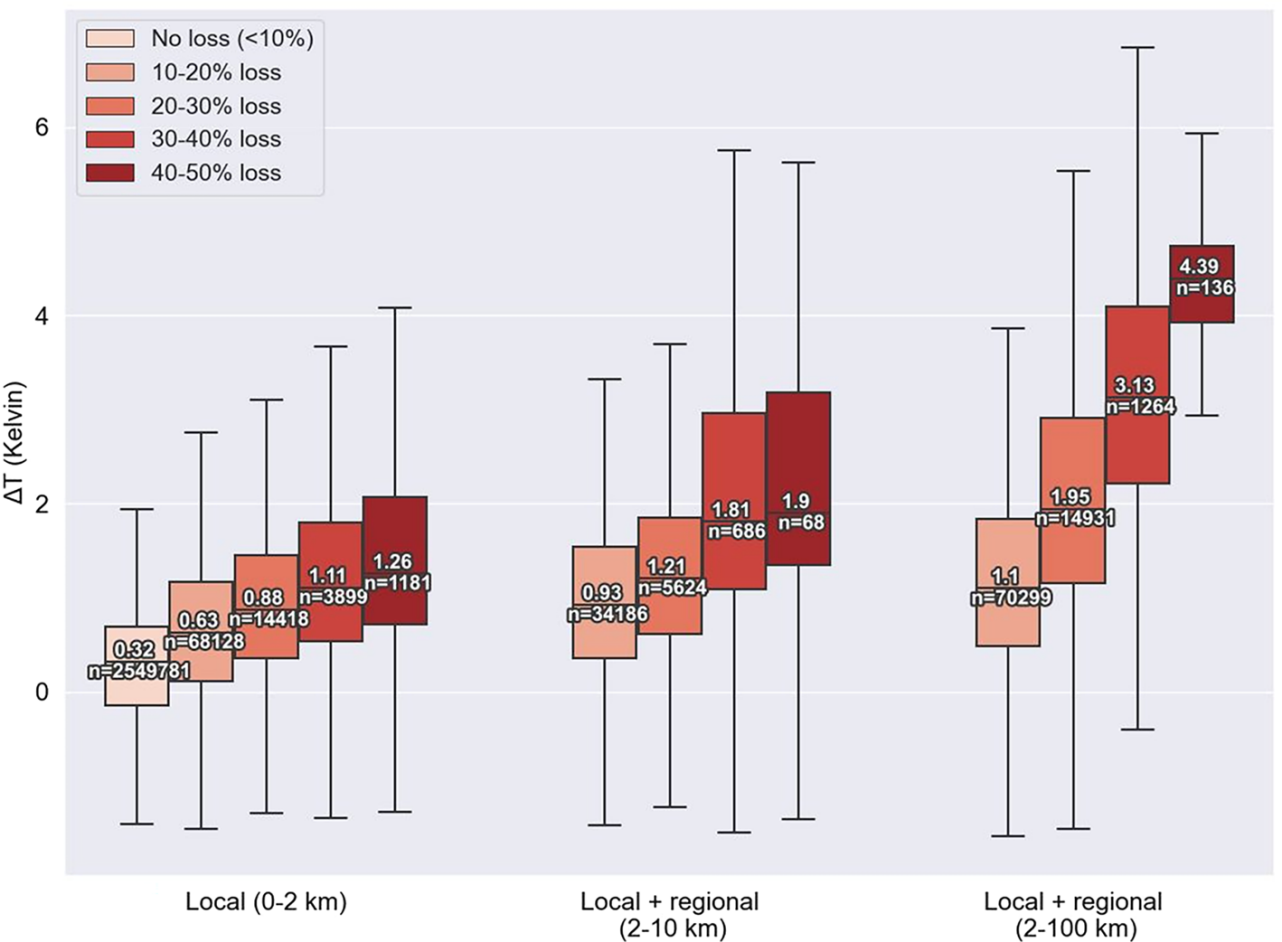
Change in observed dry season surface temperature (Δ*T*, Kelvin) as a function of forest loss at different length scales. Δ*T* is shown from left to right: Local (0–2 km) forest loss only (i.e., less <10% forest loss at regional scales from 2 to 100 km), local plus regional forest loss at 2–10 km (i.e., less <10% forest loss at regional scales from 10 to 100 km), and local plus regional forest loss at 2–100 km. Labeled central values are the median of the distribution. Figure is restricted to displaying forest loss <50%.

Fig. 2 shows observed Δ*T* for varying amounts of local and regional forest loss. In locations with little or no forest loss (<10 percentage points forest loss), median Δ*T* is 0.3 K, which we consider a background rate. Locations with 10–20% local forest loss but little regional forest loss (i.e., <10 percentage points forest loss at regional scales from 2 to 100 km), experience median warming of 0.6 K, or double the background rate. Locations with 40–50% local forest but little regional forest loss experience a median warming of 1.3 K, more than quadruple the background rate. Warming over locations with both local and regional forest loss is even greater. Locations with 10–20% local forest loss and 10–20% regional forest loss at scales of 2–10 km experience a warming of 0.9 K, triple the background rate. When both local and regional forest loss is 40–50%, median warming is 6 times the background rate (1.9 K). When the regional scale of forest loss extends to 100 km, warming is 3.5 times (1.1 K) to 14 times (4.4 K) greater for 10–20% and 40–50% forest loss, respectively. Warming exceeding 4 K that is observed over regions with strong local and regional forest loss is at the upper end of that reported in previous studies of tropical deforestation (5–13). Dry-season warming of 3 K has been reported for regions of large-scale deforestation in the southern Amazon (16). Together this suggests that large-scale forest loss leads to substantially greater warming.

### Machine Learning Prediction.

**Fig. 3.**
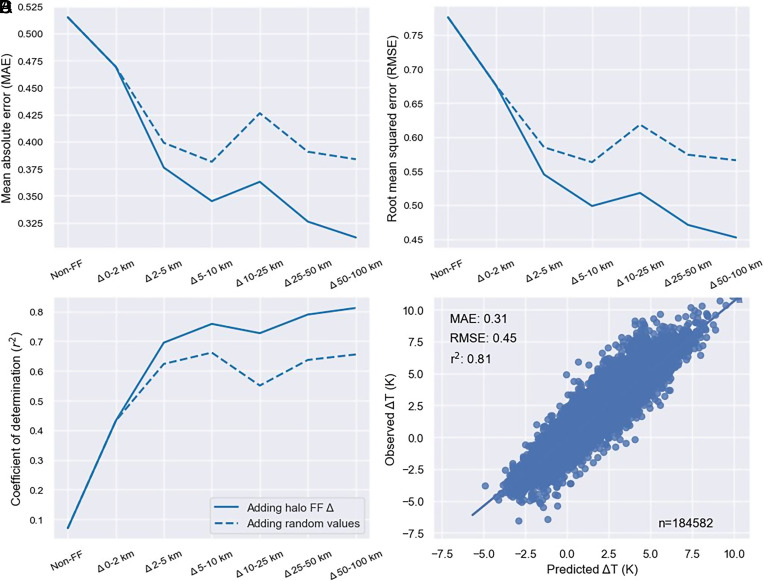
Prediction of surface temperature change due to forest loss. (*A*) Mean absolute error, (*B*) RMSE, (*C*) coefficient of determination. Model prediction metrics on the test dataset (*n* = 184,582). Simulations include no information on forest fraction loss (Non-FF), local forest loss (Δ0–2 km) only, local forest loss and information of regional forest loss at scales of 2 to 100 km added to the model incrementally (solid blue line). The blue dashed line represents adding dummy halo features incrementally populated with random values between 0 and 1. (*D*) Model prediction of Δ*T* with local and all regional forest loss features (2–100 km) included compared to observations from the test dataset.

To understand the relative roles of local and regional forest loss on Δ*T*, we developed a range of machine learning models including different aspects of local and regional forest loss. We employed the XGBoost algorithm, suitable for tabular data, using hyperparameters selected via cross-validation. Our dataset of ~3.7 million records was split into 95% training and 5% testing datasets. Models were trained and tested, including simulations with varying degrees of forest loss at different length scales. We also included a range of factors that are known to influence the temperature response to forest loss, including latitude (9), elevation (28), and distance to coast (29). Fig. 3 shows our machine learning prediction of Δ*T* using different local and regional forest loss features (*Methods*). Predictions of Δ*T* are poor when no information on forest loss is included in the model training (*MAE* = 0.52, *RMSE* = 0.78, *r   *^2^ = 0.07) (Fig. 3 *A*–*C*). Including information on local (0–2 km) forest loss improves the model, albeit with substantial scatter in predictions (*MAE* = 0.47, *RMSE* = 0.68, *r   *^2^ = 0.43). Including information on regional forest loss substantially improves the model with a consistent improvement to the model performance when information on regional forest loss at scales of 2–100 km is included (Fig. 3 *A*–*C*, solid blue line). The best prediction is found when the model includes information on forest loss out to the 100-km scale (Base model: *MAE* = 0.31, *RMSE* = 0.45, *r   *^2^ = 0.81), resulting in a model that is substantially better than the model based on local forest loss only. Adding features can artificially improve the model performance. To ensure that this was not influencing our analysis, we show that incrementally adding dummy forest fraction halos populated with random forest loss data did not improve model prediction (Fig. 3 *A*–*C*, dashed blue line). These results demonstrate that accounting for regional forest loss greatly improves Δ*T* prediction. Overall, our modeling results show that good Δ*T* prediction is only achieved when both local and regional (non-local) forest loss at length scales up to 100 km are accounted for in model training. Our results suggest that nonlocal neighboring forest cover loss is significantly contributing to observed temperature responses across the Amazon.

#### Δ*T* Prediction as a Function of Forest Loss at Local and Regional Scales.

**Fig. 4.**
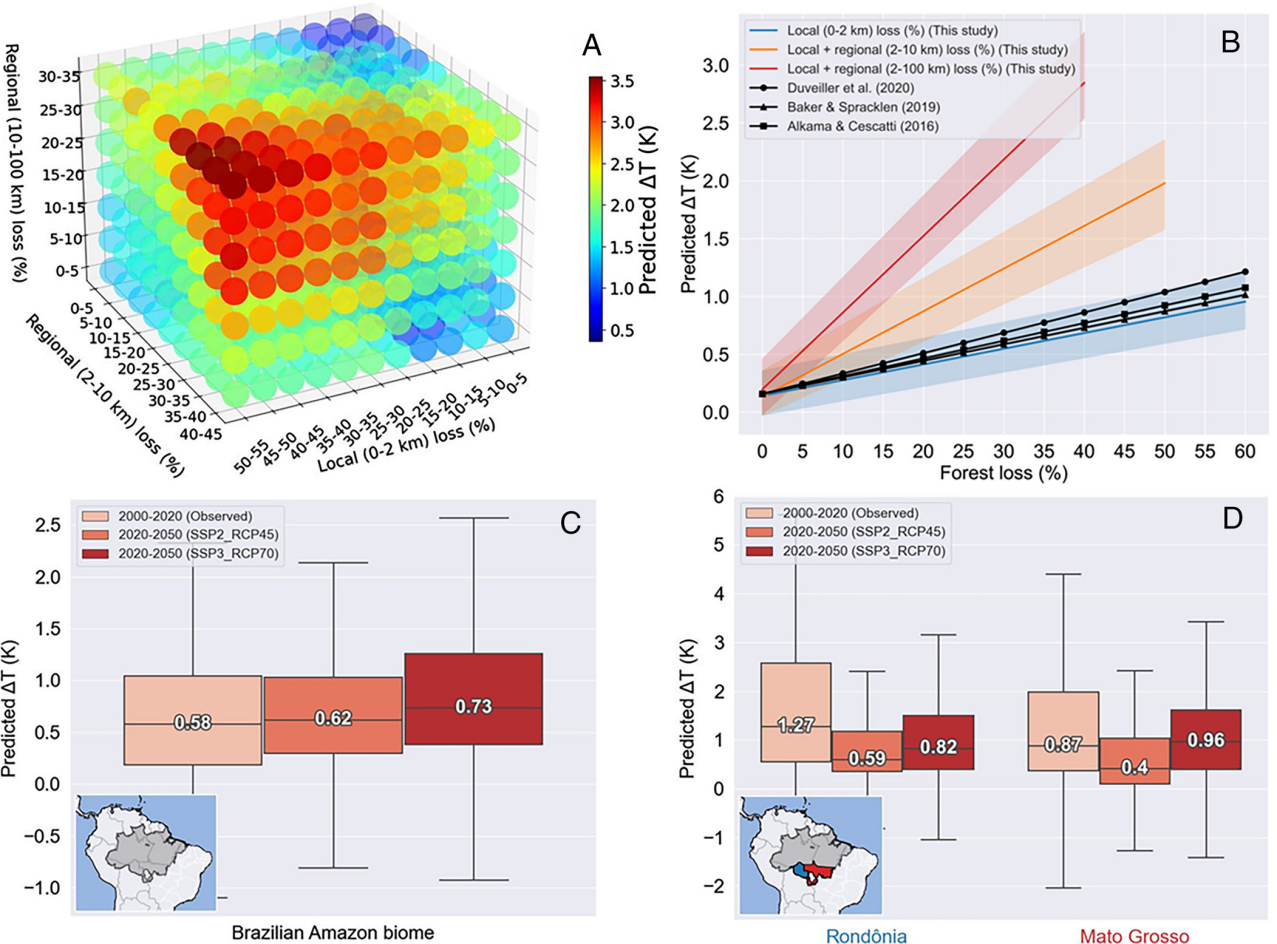
Model simulated surface temperature change (Δ*T*) due to forest loss. (*A*) Model predicted Δ*T* from 693 simulations covering the full range of local and regional forest loss. Median predicted values are calculated using dataset average values for latitude, elevation, and distance to coast. (*B*) Model predicted Δ*T* as a function of increasing local only (blue) and local plus regional (orange and red) forest loss. Observed Δ*T* as a function of increasing tropical forest loss are from three different observational studies (6, 15, 30). (*C*) Observed (2000 to 2020) and model predicted (2020 to 2050) Δ*T* for the Brazilian Amazon biome. Predicted Δ*T* for future land cover change under a middle-of-the-road scenario (SSP2_RCP45) and a strong Inequality scenario (SSP1_RCP70). Median Δ*T* values are provided on each boxplot. (*D*) Observed (2000 to 2020) and model predicted (2020 to 2050) Δ*T* for two Brazilian states located in the southern Brazilian Amazon biome: Rondônia (blue polygon) and Mato Grosso (red polygon).

Fig. 4 shows predicted Δ*T* as a function of local and nonlocal forest loss. Warming over regions with only local forest loss increases from <0.2 K over regions with less than 5% local forest loss to 0.7 K when local forest loss reaches 40% (Fig. 4*B*). In regions with both local and regional (2–10 km) forest loss, warming increases to 1.6 K when forest loss reaches 40%. When the regional extent of forest loss extends further to 100 km, warming in areas of 40% forest loss increases to 2.8 K (Fig. 4*B*). For each 10-percentage points of forest loss, warming increases by an average of 0.16 K for local forest loss only, to 0.4 K for local and regional (2–10 km) forest loss, and to 0.71 K for local and regional (2–100 km) forest loss. Our results therefore show that regional forest loss at scales up to 100 km increases the warming due deforestation by more than a factor of 4. Our modeling results provide further evidence that both local and nonlocal forest losses are contributing to the climate responses observed across the Amazon biome. Importantly, our results show that previous estimates may underestimate the warming from tropical forest loss (6–10, 15) as they do not fully account for the contribution from regional forest loss (Fig. 4*B*). Many of these studies apply a nearest neighbor or difference-in-difference approach to separate temperature changes from climate change to those from deforestation. However, such approaches may remove the regional climate signal from the analysis, implicitly assuming that it is a signal of climate variability or change rather than a regional response to deforestation as we show here. Our analysis focused on the dry season. Tropical deforestation leads to local warming across all seasons (6, 16) and future work is needed to assess the nonlocal climate impacts of Amazon deforestation outside of the dry season.

#### Warming Associated with Future Deforestation.

The future trajectory of deforestation in the Amazon depends on a wide range of local, national and global factors. We combined our model with two land cover change scenarios for Brazil to explore the impact of these different trajectories on regional climate. Both scenarios are aligned with the Shared Socioeconomic Pathways (SSPs) and Representative Concentration Pathway (RCPs) refined with regionally specific information (31). The first scenario represents a Strong Inequality scenario (SSP3_RCP7) where the Brazilian Forest Code is not respected, protected areas are not safeguarded, and there is continued expansion of paved roads which together result in rapid continued deforestation. The second scenario represents a middle-of-the-road scenario with respect for the Forest Code, some additional safeguarding for protected areas and measures to reduce negative impacts of road expansion. Widespread forest loss is predicted in both scenarios with 762,739 km^2^ of forest loss in the strong inequality scenario and with 673,066 km^2^ of forest loss in the middle-of-the-road scenario (*SI Appendix*, Figs. S1 and S2). Across the Brazilian Amazon biome, we estimate this forest loss will cause a median (mean) surface warming of between 0.62 K (0.63 K) under the middle-of-the-road scenario and 0.73 K (0.79 K) under the strong inequality scenario (Fig. 4*C*). In the southern Amazon, rapid forest loss over the period 2000–2020 resulted in strong warming (Fig. 1*D*). Stronger future warming is also simulated across the southern Amazon where projected forest loss is greatest (*SI Appendix*, Figs. S1*D* and S2*D*). In Rondônia, predicted median (mean) surface warming increases from 0.59 K (0.75 K) under the middle-of-the-road scenario to 0.82 K (0.95 K) under the strong inequality scenario. In Mato Grosso, predicted warming increases from 0.4 K (0.56 K) under the middle-of-the-road scenario to 0.96 K (1 K) under the strong inequality scenario (Fig. 4*D*). This reduced warming in the southern Amazon in the middle-of-the-road scenario is due to lower deforestation rates in this region, which are up for factor of two slower compared to the strong inequality scenario. Our analysis isolates warming due to forest loss and does not include changes due to global warming. Potential positive feedbacks and tipping points in the system (32) will not be captured in our approach so future warming may be stronger than estimated here. Our analysis is restricted to the impacts of deforestation in the Amazon. Further work is needed to explore the nonlocal climate impacts of deforestation in other tropical forest regions.

##### Implications.

The regional warming due to Amazon deforestation will have negative consequences for the 30 million people living within the Amazon basin, many of whom are already exposed to dangerous levels of heat (33). Previous studies have suggested that the combined impacts of future climate change and deforestation could expose an additional 11 million people across the Amazon to extreme heat stress (34). Increased temperatures will reduce human productivity (35) and increase all-cause human mortality (36) for already marginalized communities. Increased temperatures will also impact livestock and reduce crop yields (37), exacerbated by deforestation-induced reductions in rainfall (38, 39). Increased regional temperatures caused by deforestation will also impact carbon storage of remaining forest (40–42) and increase the risk of fire, with the resultant haze having further consequences for human health (43).

In 2020, Amazon deforestation was at the highest rate in the last decade (44) with consequent loss of biodiversity and substantial carbon emissions (45). Our analysis shows that controlling future deforestation in the Amazon would reduce future warming particularly for the southern states of Rondônia and Mato Grosso, with important social and economic benefits across the region. Forest degradation, where forests have been selectively logged or experienced fire, impacts large areas of the Amazon (46) contributing substantially to carbon emissions (47, 48). Evapotranspiration in degraded forests can be up to 34% lower compared to intact forests (49) with likely consequences for regional climate. Recovery of secondary and degraded forests can act as a carbon sink (50) and may offer additional local and regional cooling through biophysical effects. Future work is needed to analyze the regional climate impacts of forest degradation and regrowth of secondary and degraded forests (51). Identifying options for sustainable development of the Amazon without major future deforestation or degradation is crucial (52) to supporting climate adaptation and resilience of the region.

Our analysis does not provide information on the mechanisms for regional warming. Tropical deforestation leads to local warming due to reductions in the turbulent energy flux caused by reduced evapotranspiration and reduced surface roughness outweighing the cooling effects of increased surface albedo (12). These local climate changes will generate thermal, moisture, and surface pressure gradients that will alter the lateral movement of heat and moisture (3). Changes in circulation driven by deforestation will transport warmer air from surrounding regions of deforestation (53, 54) and may alter cloud cover (55) with further impacts on temperature. In a similar way, the urban heat island effect has shown to extend into surrounding rural areas (56, 57). Climate model simulations confirm that tropical deforestation leads to local warming (2, 13), although models disagree as to the relative contribution of changes in evapotranspiration, turbulent heat fluxes, and albedo to the surface temperature change (58, 59). Climate model studies separating local and nonlocal effects of deforestation show that nonlocal effects become comparable to local effects only under extensive land cover change (4), as is now the case in the southern Amazon. However, coarse resolution global climate models simulations (58) with resolutions of 0.7°–2.8°, equivalent to 70–280 km in the tropics, are unable to separate the local and nonlocal responses of deforestation that occur at the 10 to 100 km scale. Higher-resolution simulations show how Amazon deforestation can initiate mesoscale circulations (54) contributing to nonlocal effects. Convection-permitting regional climate models simulating the impacts of deforestation at a resolution of 4 km are now available (60, 61) providing new opportunities to understand the key processes driving the observed temperature response at these scales.

Our work quantifies the nonlocal (regional) impacts of deforestation on land surface temperature across the Amazon. We show that regional forest loss increases warming by more than a factor of 4 with serious consequences for the remaining Amazon forest and the people living there. We estimate that efforts to reduce deforestation could reduce the future warming experienced over the southern Amazon by 0.56 K over the next 30 y. Such findings demonstrate the contribution of tropical deforestation to regional climate warming and the urgent need for reduced deforestation and forest conservation to deliver regional climate resilience with important implications for sustainable management of the Amazon.

## Methods

### Land Cover Classification Data.

Land cover data from the MapBiomas project (62) was used, which provides annual land cover classification at 30-m resolution for the period 1985 to 2020. We used data for the year 2020.

### Rainfall Data.

We used precipitation data from Climate Hazards Group InfraRed Precipitation with Stations (CHIRPS) version 2.0 (63). The CHIRPS dataset is a blended rainfall product combining 5-y precipitation climatology, satellite observations, model simulations, and in situ observations from gage stations. Quasiglobal gridded products are available from 1981 to near-present at 0.05° spatial resolution (approx. 5 km at the equator) (63). We used monthly data spanning the period 1 January 2001 to 31 December 2020.

### Elevation Data.

We used elevation data taken from Global Multi-resolution Terrain Elevation Data (GMTED2010) (64) at 7.5-arc-second spatial resolution.

### Distance to Coast Data.

We used data on distance to the nearest coast from https://oceancolor.gsfc.nasa.gov/resources/docs/distfromcoast/.

### Land Surface Temperature Data.

We used land surface temperature (LST) data from MOD11A2 version 6 MODIS 8-d LST data at 0.01-degree resolution grid downloaded from https://www.earthdata.nasa.gov/ (variable name: LST_Day_1km). Following Li et al. (9), we excluded data where the estimated emissivity error was greater than 0.02 and where the LST error was greater than 1 K. Extensive cloud cover can reduce the spatial and temporal availability of satellite data. For this reason, we focus our analysis on the dry season when there is less cloud cover. To obtain monthly data, we aggregated by month ignoring any 8-day period where data were missing due to clouds or as a result of the quality screening process. Although Terra’s 10:30 AM local overpass time usually senses a cooler surface than Aqua with its 1:30 PM overpass, we opted to use Terra for its longer sampling period and due to lower cloud cover in the morning.

For each pixel, we analyzed LST data for the driest month identified using CHIPRS monthly rainfall data (*SI Appendix*, Fig. S3). Dry season surface temperature change (Δ*T*) was then calculated by subtracting the average surface temperature of the driest month for two periods at the end (2018 to 2020) and start (2001 to 2003) of the study period. Using 3-y averages reduces the influences of climate variability. As a final preprocessing step, we used land cover classifications in the year 2020 to remove data points that contained ≥5% fraction of water, urban, flooded forest, and wetlands in both start and end periods. In addition, we excluded pixels at elevations above 500 m to avoid significant effects of elevation on Δ*T*. Our final Δ*T* dataset comprised *n* = ~3.7 million pixels (1 km^2^ in extent) over the Amazon biome region.

### Forest Fraction Data.

Forest fraction data were taken from Global Forest Change (Source: Hansen/UMD/Google/USGS/NASA) (23) V1.8. Annual forest fraction for the period 2000 to 2020 was calculated by taking tree cover in the year 2000, defined as canopy closure for all vegetation taller than 5 m in height encoded as a percentage per output grid cell units ranging 0–100 and subjecting it to annual forest loss, defined as a disturbance from a forest to nonforest state. Forest loss from this product has user’s and producer’s accuracies of greater than 80%. Our analysis does not fully represent forest degradation or secondary forest regrowth. Average forest fraction change was calculated as the difference between average forest fraction for two periods at the start (2001 to 2003) and end of the study period (2018 to 2020). We calculated both local and nonlocal regional forest cover change at different length scales of Δ*T* locations. Local forest cover change included forest loss at distances of 0–2 km, while regional forest cover change was calculated in “halos” located 2–5 km, 5–10 km, 10–25 km, 25–50 km, and 50–100 km of Δ*T* locations (*SI Appendix*, Fig. S4). Halo analysis was conducted in Python version 3.9.7 using the Geopandas package version 0.10.2.

Large forest losses occur not only at local scales (0–2 km), but also at nonlocal regional length scales 2–10 km and 10–100 km from of Δ*T* locations. We find a 2-percentage point change in median forest cover between start (99%) and end (97%) forest fraction period at local scales (0–2 km) of Δ*T* locations (*SI Appendix*, Fig. S5). For nonlocal regional distances 2–10 km of Δ*T* locations, we find a 5-percentage point change in median forest cover between start (96.5%) and end (91.5%) periods, while we find a 7-percentage point change at regional distances 10–100 km of Δ*T* locations between start (92%) and end (85%) periods (*SI Appendix*, Fig. S5).

### Supervised Machine Learning.

We used a supervised machine learning (ML) model to predict Δ*T*. All data was regridded to 0.01° resolution. Table 1 provides details of the features used in the model including ΔT, local and regional forest cover at the start and end of the analysis period, latitude, elevation above sea level, and distance to the nearest coast. We used a gradient-boosting decision tree algorithm (XGBoost) (65) as our model of choice, well suited to the regression problem with tabular data. Briefly, the XGBoost algorithm adds decision tree models to an ensemble that are fit to correct errors made by prior decision tree models. Models are fit using a loss function and gradient descent optimization algorithm whereby the loss gradient is minimized as the model is fitted to training data (gradient boosting). The algorithm is computationally efficient and highly effective, as well as being found to dominate regression problems using tabular datasets on the Kaggle competitive data science platform (65).

**Table 1.** Description of model features

Feature name	Distance	Description	Units
Δ*T*	0 km	Target feature: Surface temperature change calculated by subtracting the average surface temperature of the driest month for two periods at the end (2018 to 2020) and start (2001 to 2003) of the study period.	Kelvin (K)
Latitude	0 km	Model feature: Latitude at Δ*T* locations.	Degrees: rescaled (0 to 1)
Elevation	0 km	Model feature: Elevation at Δ*T* locations.	Meters (m): rescaled (0 to 1)
Distance to coast	0 km	Model feature: Distance to the nearest coastline from Δ*T* locations.	Kilometers (km): rescaled (0 to 1)
Local_0–2km_change	0–2 km	Model feature: Average forest fraction change in halo 0–2 km of Δ*T* locations.	Percentage point forest fraction loss (0 to 100)
Regional_2–5km_change	2–5 km	Model feature: Average forest fraction change in halo 2–5 km of Δ*T* locations.	Percentage point forest fraction loss (0 to 100)
Regional_5–10km_change	5–10 km	Model feature: Average forest fraction change in halo 5–10 km of Δ*T* locations.	Percentage point forest fraction loss (0 to 100)
Regional_10–25km_change	10–25 km	Model feature: Average forest fraction change in halo 10–25 km of Δ*T* locations.	Percentage point forest fraction loss (0 to 100)
Regional_25–50km_change	25–50 km	Model feature: Average forest fraction change in halo 25–50 km of Δ*T* locations.	Percentage point forest fraction loss (0 to 100)
Regional_50–100km_change	50–100 km	Model feature: Average forest fraction change in halo 50–100 km of Δ*T* locations.	Percentage point forest fraction loss (0 to 100)

XGBoost hyperparameters were selected based on a fivefold cross validation grid-search approach, in addition to a manual trial and error approach (see provided Jupyter notebook python code for hyperparameters used). The dataset comprising ~3.7 million records was randomly split into training and test datasets, comprising ~3.5 million training (95%) and ~0.2 million testing (5%) records, with almost identical target (Δ*T*) distributions (*SI Appendix*, Fig. S6). Models were then trained on the training dataset and tested on the test dataset.

We conducted a number of model simulations using the XGBoost algorithm (Fig. 3). The Non-FF simulation used nonforest fraction features only (latitude, elevation, and distance to the nearest coastline), which was found to be a poor model (Fig. 3). In order to understand the sensitivity of temperature response to forest loss at difference length scales, we developed a range of models including both local and regional forest loss over progressively larger distances from 0 to 100 km. Including both local and regional forest loss substantially improved model performance (Fig. 3). Adding forest fraction loss features may artificially improve model performance, so we repeated the same sensitivity, but instead substituting forest fraction loss data with random data ranging 0 to 1, which was found not to improve model prediction. Our best model used all available features including information on forest fraction loss at both local scales of 0 to 2 km and out to regional scales of 2 to 100 km (Fig. 3).

We found that there is some collinearity between forest fraction loss features (*SI Appendix*, Fig. S7), which is a problem in that it undermines the statistical significance of independence among model features. We tested two additional models whereby the six forest fraction loss features (Table 1) were replaced by exact copies of either the local forest loss feature (0–2 km) or by the outermost regional forest loss feature (50–100 km). Our reasoning was such that if collinearity was important, using multiple copies of either forest loss feature would produce a model that was just a good as the best model using the correct forest fraction loss data. However, we found that these two models were inferior when predicting subsets of the test dataset focusing on data points experiencing different levels of local and regional loss (*SI Appendix*, Fig. S8). These results suggest that these collinearities are not important because good model prediction requires information on both local and regional forest loss.

Finally, we deployed a linear least squares regression model to compare the results from the XGBoost algorithm under the best simulation using all available features. We found that using a linear model resulted in poor model prediction (*SI Appendix*, Fig. S9) justifying the supervised machine learning algorithm used here.

We used the model combined with observed forest loss to simulate the 2000–2020 warming across the Amazon. We did this separately for locations where we had observations of as well as across the whole Brazilian Amazon Biome. We found median (mean) warming of 0.58 K (0.78 K) over locations with observations and 0.47 K (0.63 K) over the whole biome.

### Forest Cover Change Scenarios in 2050.

We use a new set of land cover change scenarios for the Brazilian Amazon biome to 2050 (31) to predict future forest loss across the region. The scenarios are aligned with SSPs and RCPs and consider a balance between global forest loss drivers (such as GDP growth, population growth, per capita consumption of agricultural products, international trade policies, and climatic conditions) and local factors driving deforestation (such as land use, agrarian structure, agricultural suitability, protected areas, distance to roads, and infrastructure projects) (31). We consider two scenarios: a middle-of-the-road narrative (SSP2_RCP4.5) and a strong inequality scenario (SSP3_RCP7.0). Both scenarios exhibit considerable forest cover loss by 2050 with greater wide-spread forest loss in SSP3_RCP7.0 (*SI Appendix*, Figs. S1 and S2). Data for each scenario is provided on a 10 x 10 km grid in NetCDF format (https://zenodo.org/record/5123560#.Y9zmhT3P2Ls) at 5-y increments from 2015 to 2050. We regridded the data by assigning the same forest fraction value from the native resolution within a 0.01-degree resolution grid (approx. 1 km at the equator) for each scenario to obtain forest loss to 2050. Finally, we estimate warming associated with each scenario across the whole Brazilian Amazon Biome between 2020 and 2050.

## Supplementary Material

Appendix 01 (PDF)

## Data Availability

Data and code that support the findings of this study are available at http://archive.researchdata.leeds.ac.uk/id/eprint/1085 (66). Dataset and code data have been deposited in Amazon deforestation causes strong regional warming (http://archive.researchdata.leeds.ac.uk/id/eprint/1085) (66).
