# Supplementary material for: Amazon deforestation causes strong regional warming
Source: Proc Natl Acad Sci U S A. 2023 Oct 30;120(45):e2309123120. doi: 10.1073/pnas.2309123120 (PMC10636322; doi:10.1073/pnas.2309123120)
Supplement: Supplementary file 1 — Appendix 01 (PDF) [file pnas.2309123120.sapp.pdf]

Supporting Information for

Amazon deforestation causes strong regional warming

Edward W. Butt[1*], Jessica C. A. Baker[1], Francisco G. Silva Bezerra[2], Celso von Randow[2], Ana P. D. Aguiar[2, 3] and Dominick V. Spracklen[1]

[1]School of Earth and Environment, University of Leeds, Leeds, UK
[2]National Institute for Space Research (INPE), São José dos Campos, Brazil.
[3]Stockholm Resilience Centre, Stockholm, Sweden.

*Correspondence to Edward W. Butt: e.butt@leeds.ac.uk

Contents of this file

Figures: Supplementary Figures 1 to 9

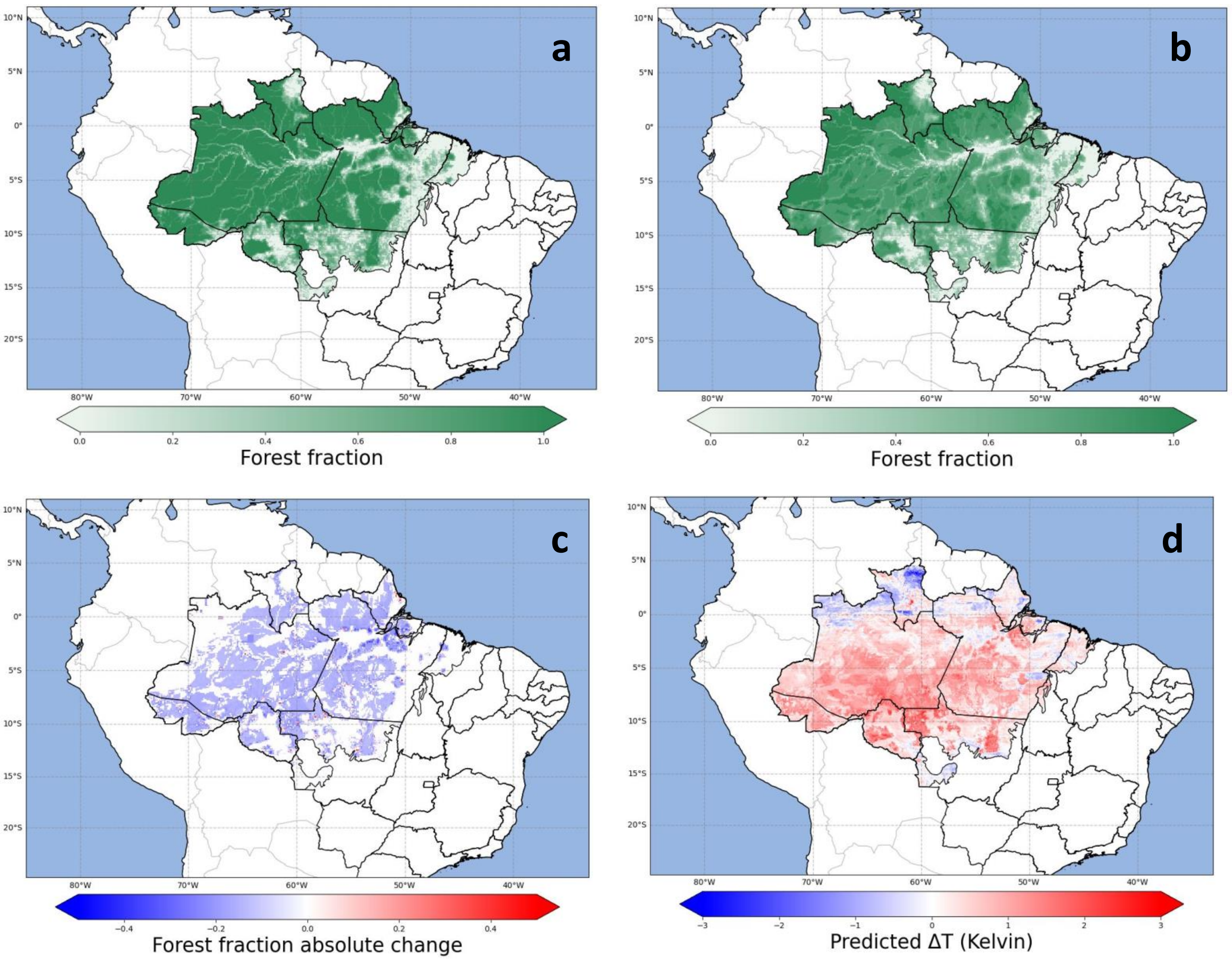

Supplementary figure 1: Predicted forest fraction in (**a**) 2020 and (**b**) 2050 under the SSP2_RCP45 scenario. (**c**) Absolute change in forest fraction (2020 - 2050). (**d**) Predicted surface temperature change (ΔT) due to forest loss by between 2020 and 2050.

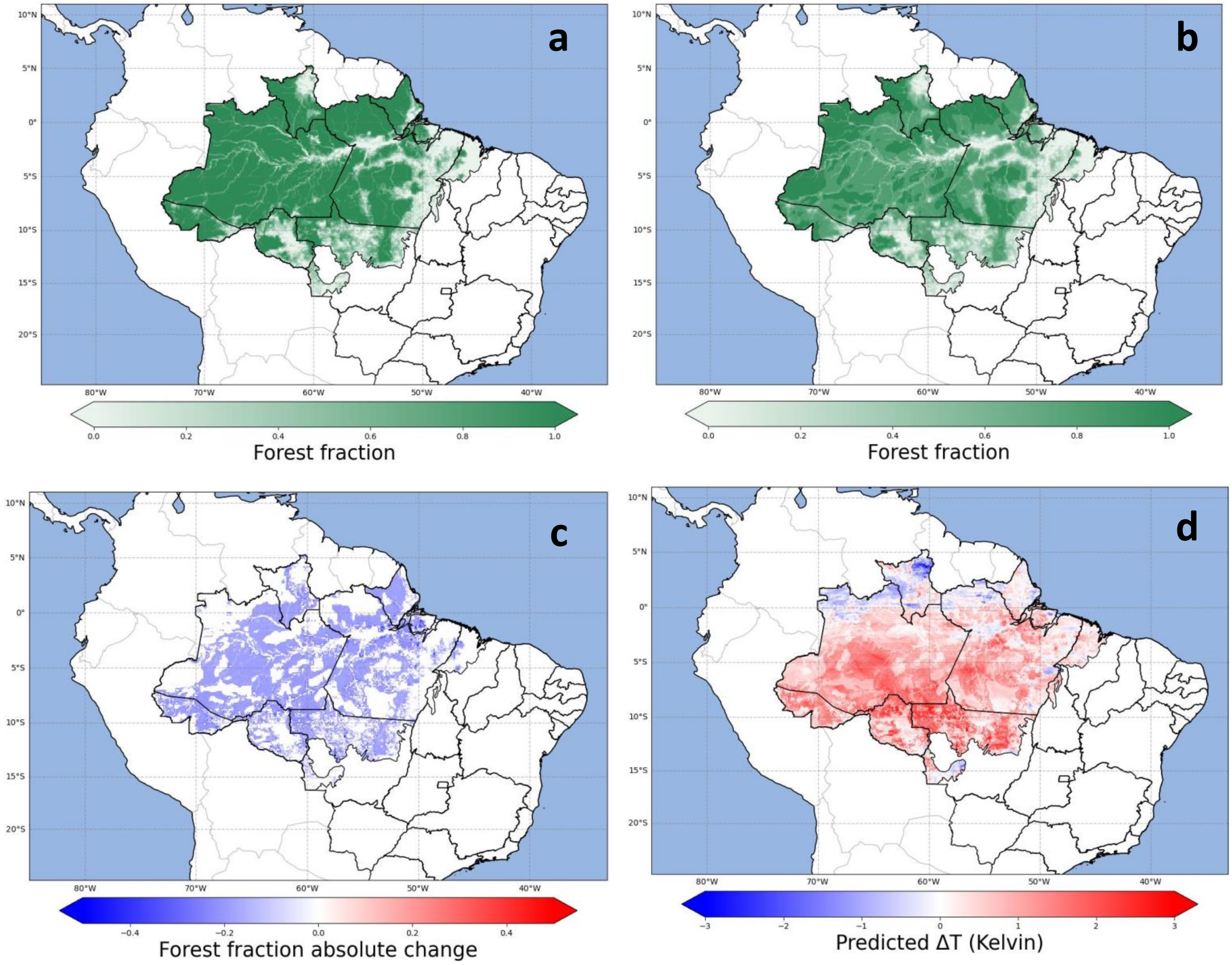

Supplementary figure 2: Predicted forest fraction in (**a**) 2020 and (**b**) 2050 under the SSP3_RCP70 scenario. (**c**) Absolute change in forest fraction (2020 - 2050). (**d**) Predicted surface temperature change (ΔT) due to forest loss by between 2020 and 2050.

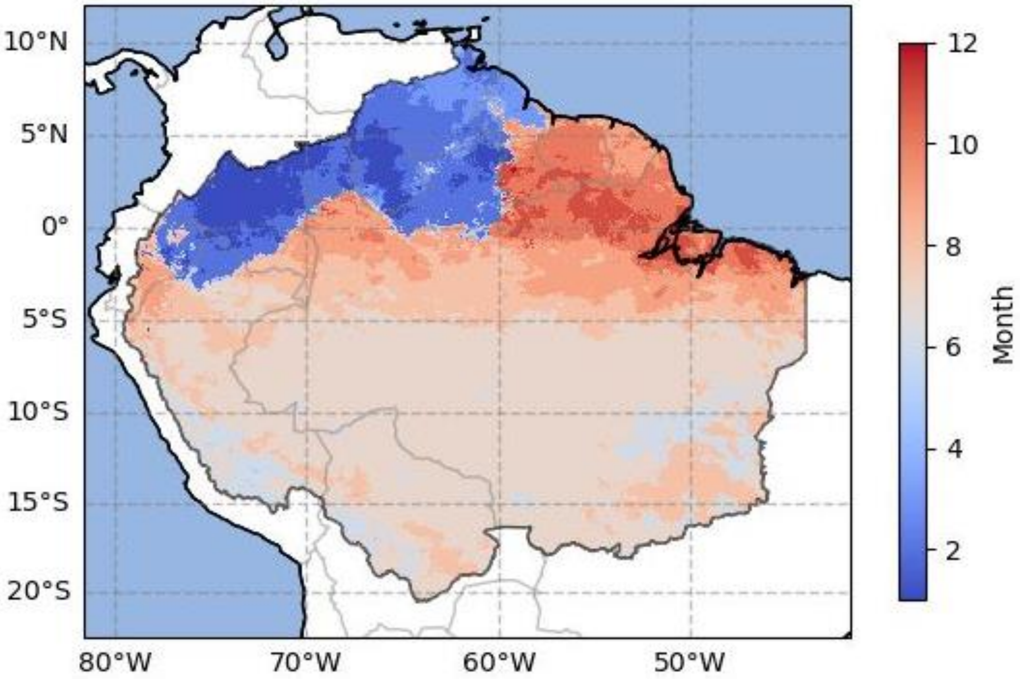

Supplementary figure 3: Driest month at 0.01° spatial resolution based on a rounded average of monthly rainfall minimums from CHIRPS at 0.05° resolution for the period 2001 to 2020.

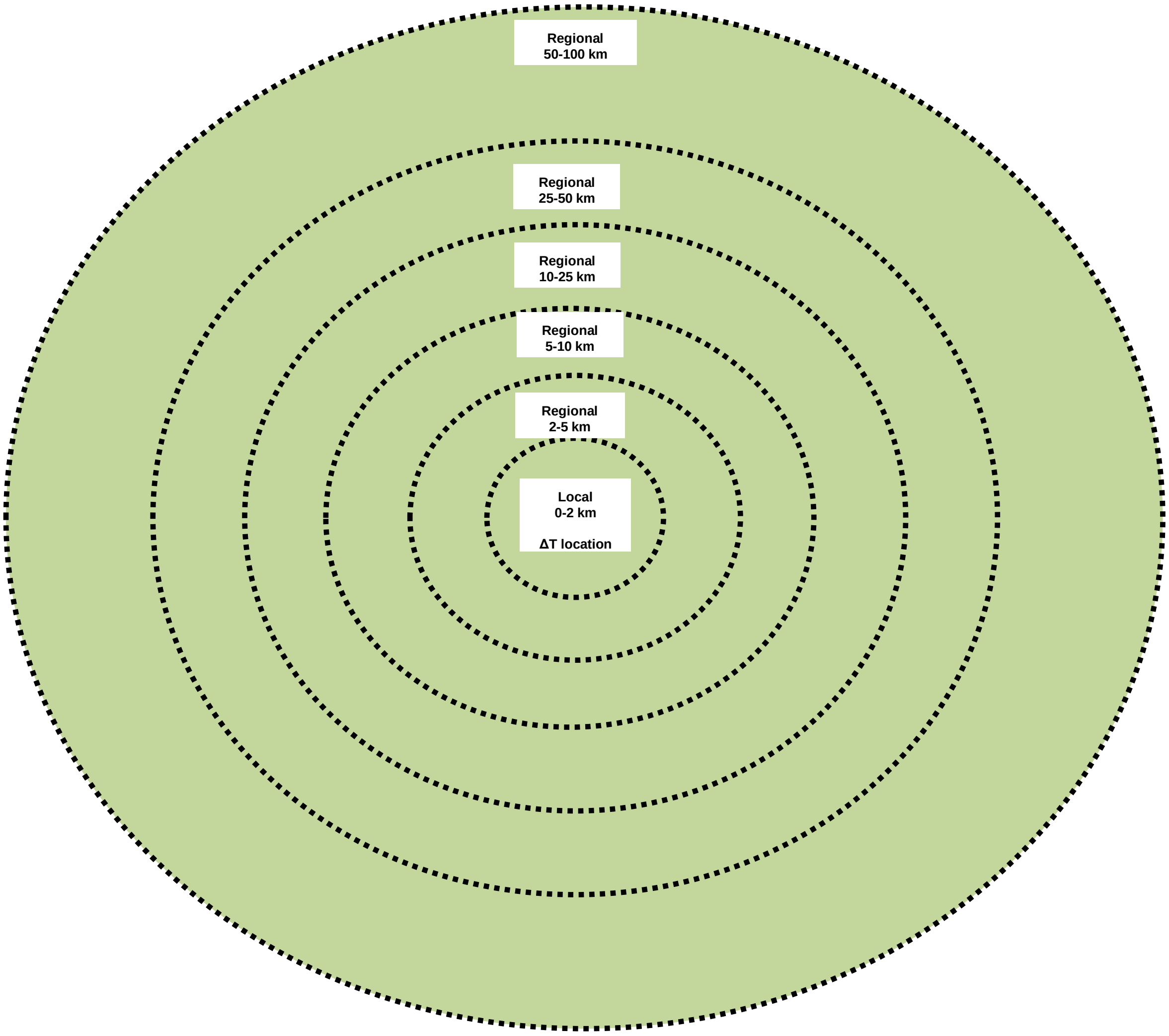

Supplementary figure 4: Pictorial representation of forest fraction loss features at increasing length scales of ΔT locations. See Table 1 for more information data features. Diagram not to scale.

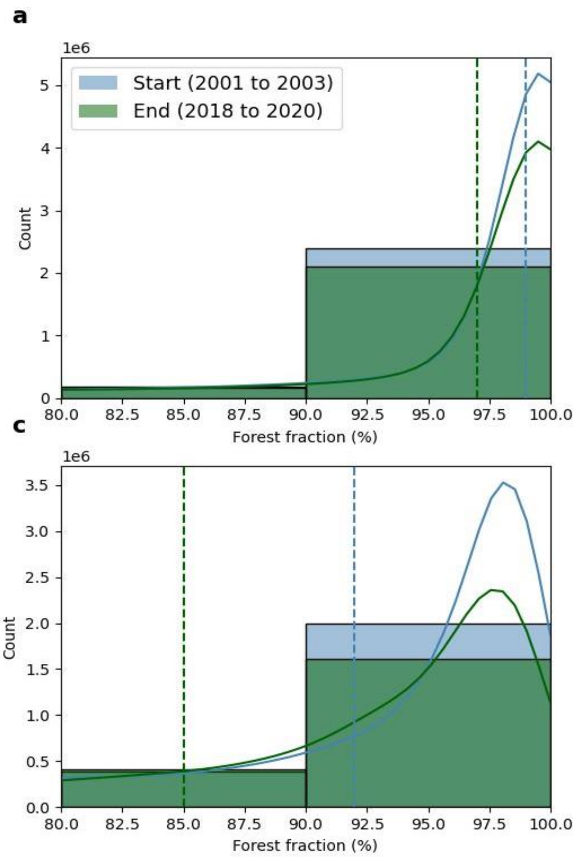

Supplementary figure 5: Histogram showing (a) local, (b) regional (2-10 km), and (c) regional (10-100 km) start (blue) and end (green) forest fraction of ΔT locations. Dashed lines represented median start and end forest fraction

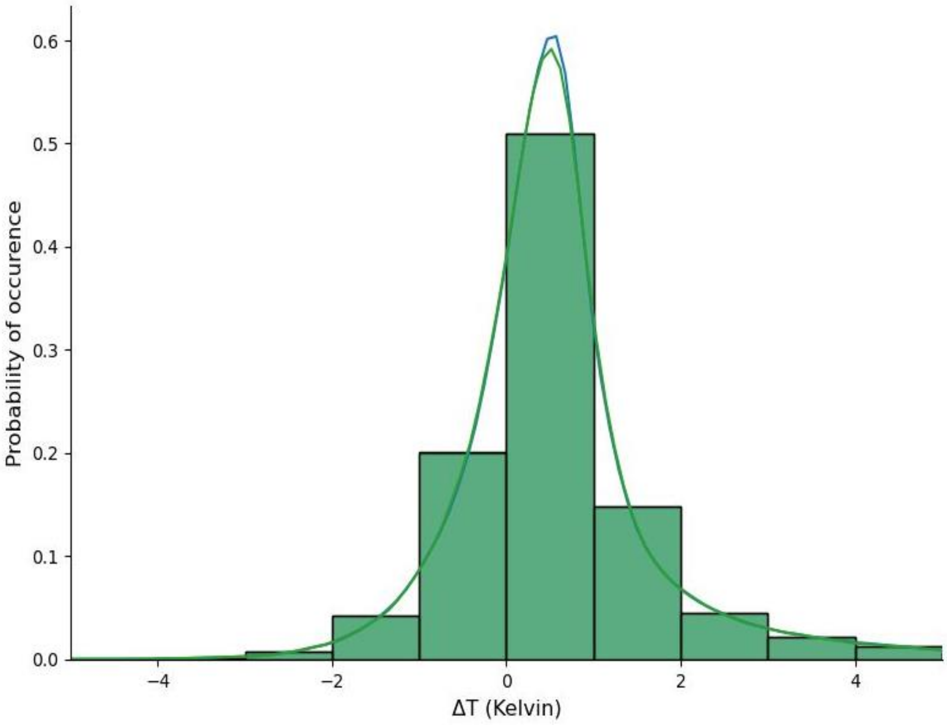

Supplementary figure 6: Histogram showing ΔT distribution for training (blue) and test (green) datasets.

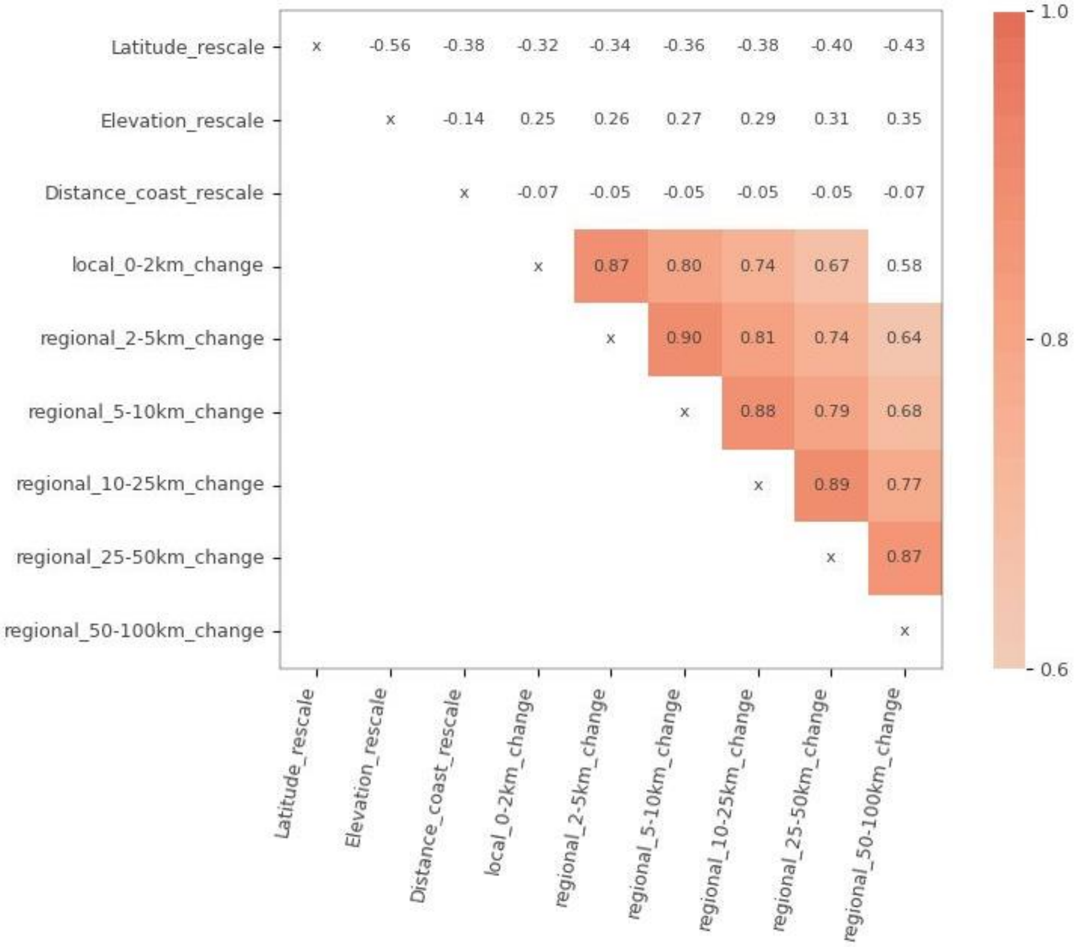

Supplementary figure 7: Spearman's rank-order correlation matrix between all pairs of model features.

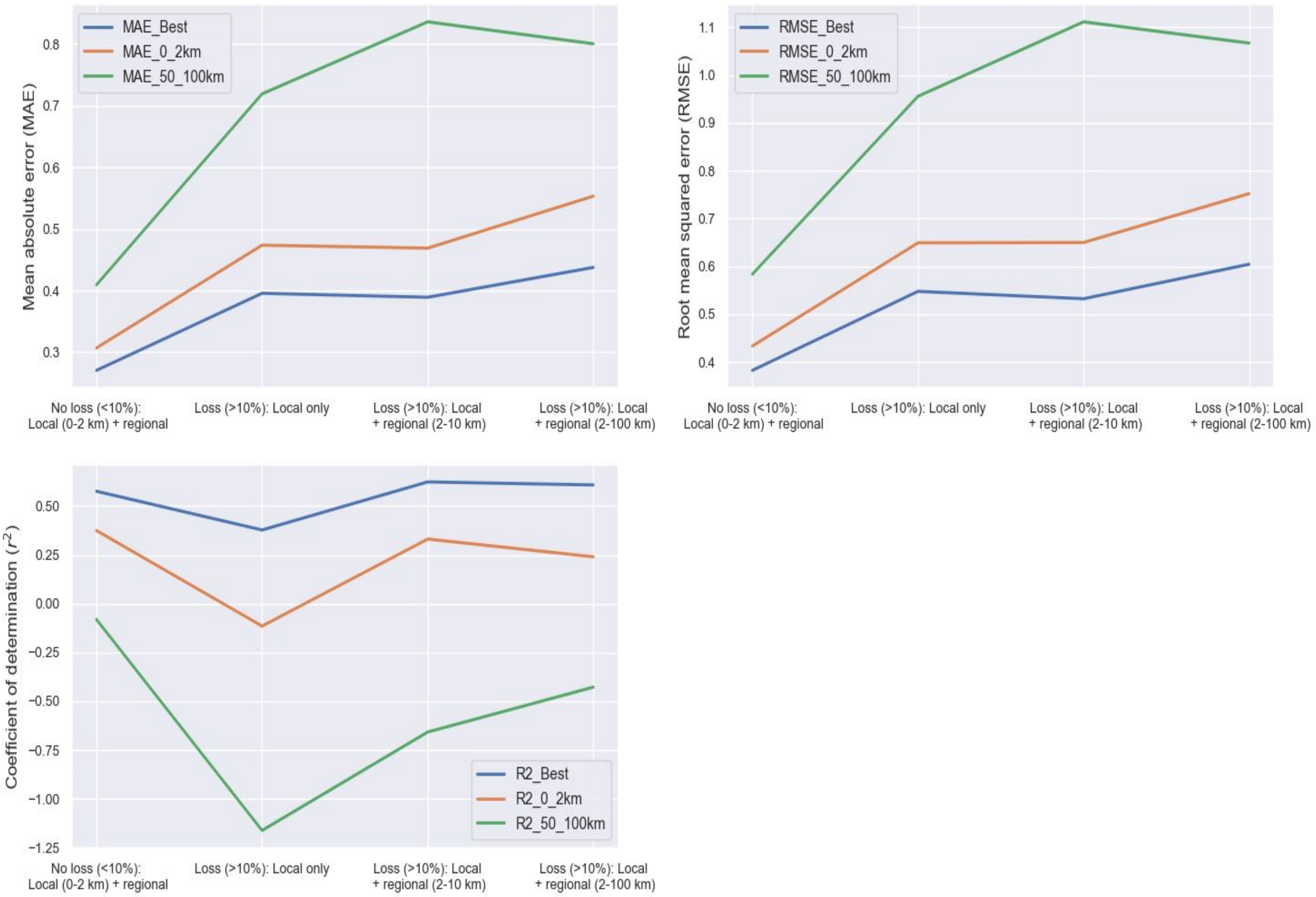

Supplementary figure 8: Two additional model simulation compare with the best model (*_Best) whereby the six forest fraction loss features (see Table 1) were replaced by exact copies of either the local forest loss feature (0-2 km, *_0_2km) or by the outer most regional forest loss feature (50-100 km, *_50_100km). We found that these two models were inferior when predicting subsets of the test dataset focusing on data points experiencing different levels of local and regional loss compared to the best model.

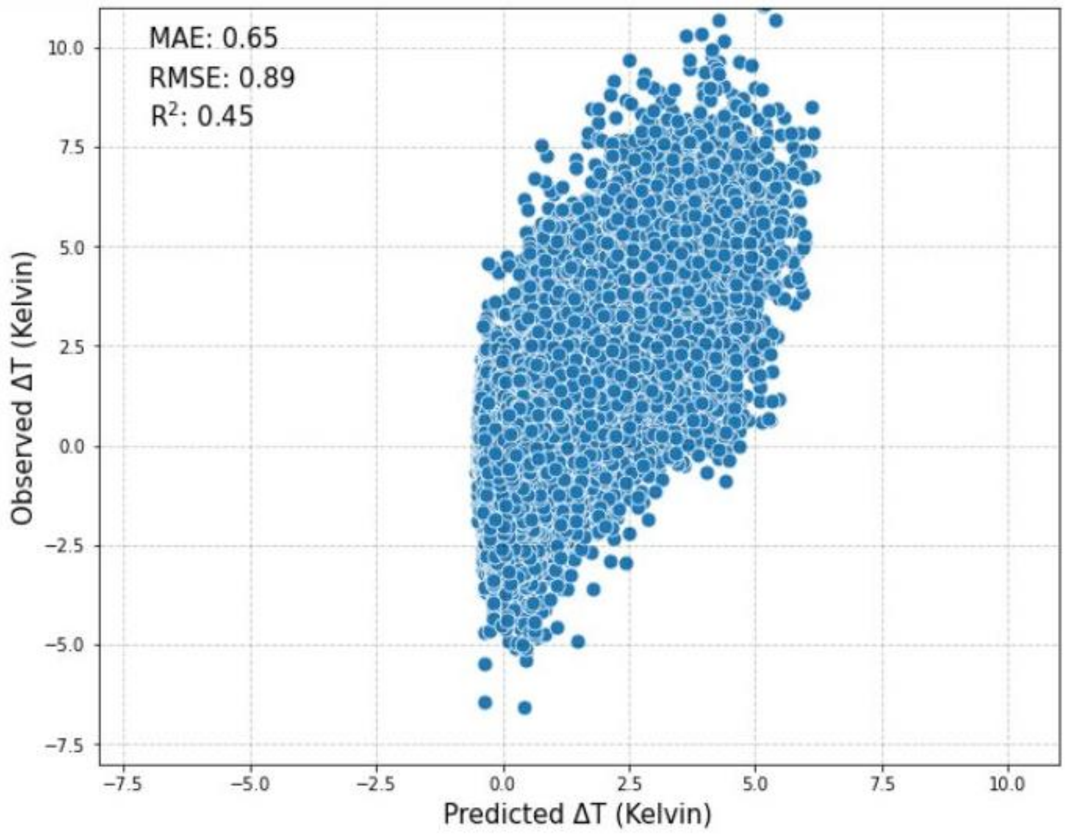

Supplementary figure 9: Least squares linear model ΔT prediction on the test dataset (n=184582).
